# Estimation of Surface Area and Volume of a Nematode from Morphometric Data

**DOI:** 10.1155/2016/6767538

**Published:** 2016-03-27

**Authors:** Simon Brown, Kevin C. Pedley, David C. Simcock

**Affiliations:** ^1^Deviot Institute, Deviot, TAS 7275, Australia; ^2^School of Human Life Sciences, University of Tasmania, Locked Bag 1320, Launceston, TAS 7250, Australia; ^3^Institute of Food, Nutrition and Human Health, Massey University, Private Bag 11222, Palmerston North, New Zealand; ^4^Faculty of Medicine and Bioscience, James Cook University, Cairns, QLD 4870, Australia

## Abstract

Nematode volume and surface area are usually based on the inappropriate assumption that the animal is cylindrical. While nematodes are approximately circular in cross section, the radius varies longitudinally. We use standard morphometric data to obtain improved estimates of volume and surface area based on (i) a geometrical approach and (ii) a Bézier representation of the nematode. These new estimators require only the morphometric data available from Cobb's ratios, but if fewer coordinates are available the geometric approach reduces to the standard estimates. Consequently, these new estimators are better than the standard alternatives.

## 1. Introduction

The physiological activities of nematodes, such as respiration or excretion/secretion, have been expressed on various bases, including wet or dry weight [1], protein [2], surface area [3], and nematode number [4]. Each of these has a distinct significance. Dry weight is indicative of the total amount of material comprising the nematode, including that which is metabolically inactive. Wet weight has the added complication that there is residual water outside the animal which is not related to its physical structure. Protein, commonly used by biochemists, depends on the specific assay employed, since protein properties and nonprotein contaminants interfere with the chemistry of each assay differently [5]. Surface area is especially relevant to transport studies [3, 6], but it is difficult to estimate. Nematode number is relatively easily estimated, but differences in the size of individuals can obscure the significance of the data reported.

Nematodes vary greatly in morphology between species [7–9] and life cycle stages, and the morphology of parasitic nematodes can depend on the host [10]. For example, *L*_3_ *Teladorsagia circumcincta* essentially has a cylindrical body tapering to relatively pointed ends, the posterior tip being more pointed than the anterior [8, 11]. Whereas the adult male, for example, is substantially larger and has a bursa at the posterior that is wider than rest of the body of the nematode, other species have a posterior reminiscent of a needle.

The morphometry of nematodes is often based on the length and width of the body [8, 12, 13] or of specific anatomical features [14]. Nematodes are frequently described as cylindrical; among many examples, Sims et al. [3, 6] estimated the surface area and volume of* Ascaris suum* and adult* Haemonchus contortus* from the length and width assuming the nematode to be cylindrical. Andrássy's [15] volume estimate is also based on that of a cylinder,

(1) $$\begin{array}{c} V_{A}\approx \frac{4}{5}\pi L{\left(\;\frac{D}{2}\;\right)}^{2}, \end{array}$$

but includes a correction factor. Holovachov [16], citing the work of Tsalolikhin, estimated the volume of a nematode using

(2) $$\begin{array}{c} V_{T}=\frac{1}{24}\left[\;\pi L\left(\;d^{2}+dD+D^{2}\;\right)+\pi LD^{2}\;\right], \end{array}$$

where *L*, *D*, and *d* are the length, maximum diameter, and labial region diameter, respectively. Of course, nematodes are none of these shapes, so a means of representing the variety of morphologies that better describes the surface area and volume is required. Here we provide two complementary approaches to this problem based on common morphometric measurements.

## 2. Common Morphometric Measurements

The commonly used morphometric measurements [17–19] relate the distance between the anterior of the nematode and particular anatomical features (*L*_*i*_) and the diameter (*D*_*i*_) of the nematode at each point. While Cobb [19] described his approach as a “formula,” as Van Cleave [20] suggested, it is really just a short-hand notation for particular ratios. The specific anatomical points defined by Cobb [19] are the base of the pharynx or the buccal cavity (*L*_1_ and *D*_1_), the nerve ring (*L*_2_ and *D*_2_), the end of the oesophagus or base of the neck (*L*_3_ and *D*_3_), the vulva in females or middle of the nematode in males (*L*_4_ and *D*_4_), and at the anus (*L*_5_ and *D*_5_). Cobb [19] reports the overall length of the nematode (*L*) and *l*_*i*_ (= *L*_*i*_/*L*) and *d*_*i*_ (= *D*_*i*_/*L*) as a percentage. While these values do not provide a complete description of the nematode, they do provide a means of generating the characteristic shape of the nematode as we describe here.

The more commonly used de Man indices [17–19] are the ratio of the length to the greatest diameter (*a*), the ratio of the length to the length of the oesophagus (*b*), the ratio of the length to the length of the tail (*c*), and the ratio of tail length to radius at the anus (*c*′). These are usually supplemented with other measurements, but what is reported varies considerably. Of course, some of Cobb's ratios can be expressed in terms of the de Man indices:

(3) l3=b−1,d4≈2a−1,l5=1−c−1,d5=2cc′

and in some cases other measurements make it possible to calculate more of Cobb's ratios. Interestingly, some authors confuse de Man indices with Cobb's ratios [22, 23].

While Cobb's ratios are more comprehensive than the de Man indices and they are still used [24–27], the latter are in more widespread use. Fracker [28] suggested that Cobb had never substantiated the consistency of his ratios and pointed out that it is sometimes difficult to identify some of the required anatomical features. Such problems may represent an impediment to the use of Cobb's ratios for taxonomic purposes, but there is no such barrier to their use in modeling the geometry of a nematode. In fact, providing the measurement points that are distributed appropriately, it may not matter particularly that they are made consistently. However, the inadequacy of the de Man indices is indicated by the frequency with which they are supplemented by other measurements.

## 3. A Simple Geometric Model

For each nematode the volume (*v*) can be thought of comprising the volumes of the head (*v*_*H*_), core (*v*_*C*_), and tail (*v*_*T*_),

(4) $$\begin{array}{c} v=v_{H}+v_{C}+v_{T} \end{array}$$

and a surface area *a*_*s*_ made up of the surface areas of the sides of the head (*a*_*sH*_), core (*a*_*sC*_), and tail (*a*_*sT*_),

(5) $$\begin{array}{c} a_{s}=a_{sH}+a_{sC}+a_{sT}. \end{array}$$

The surface area of the ends of the core must match the surface area of the base of the head and of the tail as appropriate. Of course, the length of these geometrical elements must sum to the length of the nematode.

While nematodes are often treated as cylindrical, a slight elaboration of this geometrical model is helpful. The frustum of a cone has a volume and surface area given by

(6) v=πh3r12+r1r2+r22,

(7) as=πr1+r2r1−r22+h2+πr12+r22,

respectively, where *r*_1_ and *r*_2_ are the radii of the base and the top of the frustum (so *r*_1_ ≥ *r*_2_) and *h* is the height. It is easy to see that if *r*_2_ = *r*_1_, then the expressions reduce to those for the volume and surface area of a cylinder. On the other hand, if *r*_2_ = 0, they reduce to those of a cone. In two dimensions, as a nematode appears under the microscope, the perimeter and area of the projection of the frustum are

(8) p=2r1+r2+2r1−r22+h2,

(9) a=r1+r2h,

respectively. Applying these expressions to nematode morphology provides a flexible and simple geometrical approach that would incorporate both the standard cylindrical model and Tsalolikhin's estimator (2).

The structure defined by Cobb's ratios can be viewed as the sum of six separate geometrical elements:

(10) $$\begin{array}{c} v=\overset{6}{\underset{i=1}{\sum }}v_{i}, \end{array}$$

corresponding to those defined by the coordinates implicit in the ratios. The surface area is the sum of the corresponding surface areas less twice the surface area of circles corresponding to the interfaces between these elements:

(11) $$\begin{array}{c} a_{s}=\overset{6}{\underset{i=1}{\sum }}{a_{s}}_{i}-2\pi \left(\;r_{12}^{2}+r_{23}^{2}+r_{34}^{2}+r_{45}^{2}\;\right), \end{array}$$

similarly for the projected area (*a*) and the perimeter (*p*).

As a cone and a cylinder are simply particular cases of the frustum of a cone (10)-(11), the corresponding expressions for *a* and *p* can be written:

(12) p=2r0+rn+∑i=0n−1ri+1−ri2+li+1−li2,

(13) a=∑i=0n−1ri+ri+1li+1−li,

(14) as=πr02+rn2+∑i=0n−1ri+ri+1ri+1−ri2+li+1−li2,

(15) v=π3∑i=0n−1ri2+riri+1+ri+12li+1−li.

Clearly, (12)–(15) can be extended easily to more than *n* = 6 distinct geometric elements to obtain the natural expressions:

(16) Plimn→∞ ⁡p=2r0+rn+∫0Lds,

(17) Alimn→∞ ⁡a=∫0L2r+drdl=2∫0Lr dl,

(18) Slimn→∞ ⁡as=πr02+rn2+∫0L2r+drds=πr02+rn2+2∫0Lr ds,

(19) Vlimn→∞ ⁡v=π∫0Lr2dl,

since ∫*dr* *dl* = 0. Here, *ds* and *dl* are line elements taken along the surface and midline, respectively, of the nematode and *r* varies along the length of the nematode. In effect, Robinson [29] used a discrete version of (19) in his calculations.

## 4. Least Squares Estimates of the Bézier Representation

### 4.1. Bézier Curve Background

The Bézier curve associated with *n* + 1 points *P*_0_, *P*_1_,…, *P*_*n*_ is given by

(20) $$\begin{array}{c} C\left(\;t\;\right)=\overset{n}{\underset{i=0}{\sum }}P_{i}B_{i,n}\left(\;t\;\right), \end{array}$$

where *t* ∈ [0,1] and *B*_*i*,*n*_(*t*) is a Bernstein polynomial given by

(21) $$\begin{array}{c} B_{i,n}\left(\;t\;\right)=\left(\begin{array}{c} n\\ i \end{array}\right)t^{i}{\left(\;1-t\;\right)}^{n-i}. \end{array}$$

Of course, (20) can be written as **C** = **B** **P** and if **P** is known, then (20) can be used to calculate the Bézier curve. The least squares estimate of **P** is

(22) $$\begin{array}{c} P={\left(\;B^{\prime }B\;\right)}^{-1}B^{\prime }C \end{array}$$

[30], where **C** is a matrix containing the coordinates of the morphometric data and, if necessary to adjust for the importance of particular morphometric coordinates, this can be rewritten as

(23) $$\begin{array}{c} P={\left(\;B^{\prime }WB\;\right)}^{-1}B^{\prime }WC, \end{array}$$

where **W** is a diagonal matrix of weights.

The parametric representation of the nematode in **C**(*t*) can be used to provide an estimate of *P*, *A*, *S*, and *V* using well known expressions [31]:

(24) P=2∫αβdydt2+dxdt2dt,A=2∫αβytdxdtdt,S=4π∫αβytdydt2+dxdt2dt,V=π∫αβyt2dxdtdt.

In implementing these calculations it is useful to recall that the derivative of the Bernstein polynomial (21) is

(25) $$\begin{array}{c} \frac{d}{dt}B_{i,n}\left(\;t\;\right)=n\left(\;B_{i-1,n-1}\left(\;t\;\right)-B_{i,n-1}\left(\;t\;\right)\;\right), \end{array}$$

so that the derivative of (20) is

(26) $$\begin{array}{c} \frac{d}{dt}C\left(\;t\;\right)=n\overset{n}{\underset{i=0}{\sum }}P_{i}\left(\;B_{i-1,n-1}\left(\;t\;\right)-B_{i,n-1}\left(\;t\;\right)\;\right). \end{array}$$

### 4.2. Application to Nematode Morphology

As an explicit numerical example, the morphometric data provide 5 coordinates defined as a fraction of the length of the nematode which we supplement with two coordinates (0, 0) and (1, 0), so in (21) *n* = 6, *i* = 0,1,…, 6, and *t* = 0,1/6,2/6,…, 6/6. From this

(27) B=100000015625466563125777631251555262511664125155525777614665664729642438024316072920243424317291643321564516156433216417294243202431607298024364243647291466565777612515552625116643125155523125777615625466560000001,

which we reproduce here because it applies to every case for which there are 7 morphometric coordinates. It is clear from (20) and **B** that **C**(0) = **P**_0_ and **C**(1) = **P**_6_. Cobb [21] gives morphometric data for* Aplectus antarcticus* from which, including the supplementary coordinates,

(28) $$\begin{array}{c} C^{\prime }=\frac{1}{100}\left(\begin{array}{ccccccc} 0 & 0.1 & 12.6 & 21 & 51 & 87 & 100\\ 0 & 0.4 & 1.1 & 1.15 & 1.2 & 0.95 & 0 \end{array}\right), \end{array}$$

where the upper row of numbers represents the relative position along the length of the nematode and the lower row is the corresponding relative radius (note that Cobb [21] specifies the diameter rather than the radius). Substituting these into (22) yields estimates of the Bézier control points

(29) $$\begin{array}{c} P^{\prime }=\frac{1}{100}\left(\begin{array}{ccccccc} 6.743\times 10^{-13} & -57.417 & 151.512 & -155.854 & 127.245 & 87.37 & 100\\ 1.701\times 10^{-14} & -1.543 & 5.829 & -3.422 & 3.969 & 0.722 & 5.088\times 10^{-16} \end{array}\right) \end{array}$$

and writing (20) explicitly,

(30) Ctxtyt=P01−t6+6P11−t5t+15P21−t4t2+20P31−t3t3+⋯+15P41−t2t4+6P51−tt5+P6t6,

where **P**_*i*_ is the* i*th row of **P**. As **C**(*t*) is expressed in relative units it can be converted into dimensional form by multiplying by *L*.

**Figure 1:**
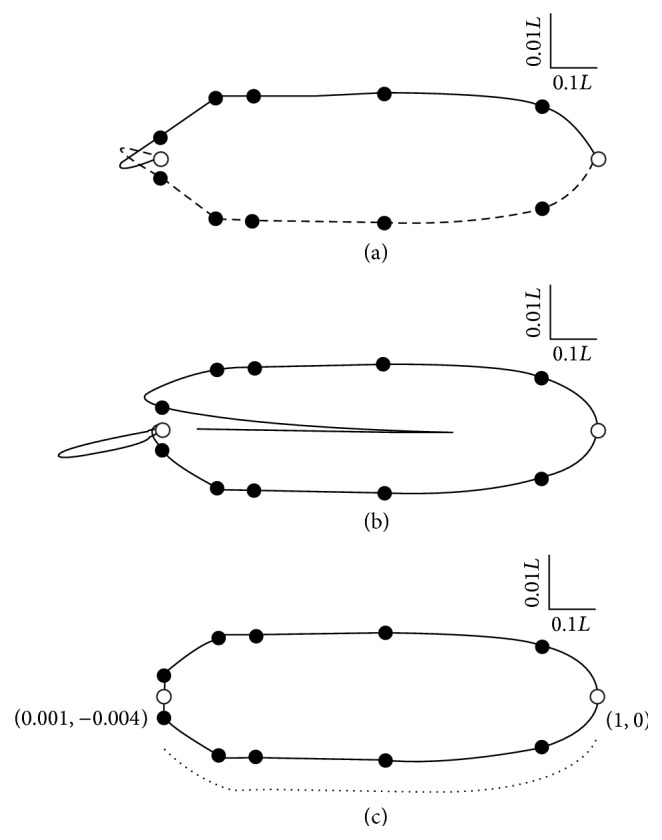
The development of the unweighted Bézier representation of* Aplectus antarcticus.* The morphometric data of Cobb [21] (●) and two supplementary coordinates (○, (0, 0) and (1, 0)) are shown. The 7-coordinate version of the unweighted Bézier representation (27)–(30) is the solid line in (a), which is reflected along the horizontal axis to give the dashed line in (a). The extended 13-coordinate Bézier representation in (b) is explained in the text, as is the edited extended representation (c) that is derived from it. The dotted line in (c) represents the region from (0.001, −0.004) to (1, 0) that is reflected about the horizontal axis to form the upper boundary. Note that the vertical and horizontal dimensions differ in scale by a factor of 10 relative to the overall length of the nematode (*L* = 0.6 mm).

Plotting *y*(*t*) against *x*(*t*) (30) yields the Bézier curve in the upper half of Figure 1(a). Since the nematode is symmetrical the lower boundary is just −*y*(*t*) against *x*(*t*), which is also shown in Figure 1(a). At least two features of the form defined by (30) are inappropriate: at the anterior end the Bézier curve forms a pair of loops and at the posterior end *dy*/*dx* approaches zero. The former is a common feature of polynomial interpolation through more than a small number of points [32]. The latter is overcome by fitting a Bézier curve clockwise through the coordinates on both sides of the nematode (Figure 1(b)). This 13-coordinate extended representation is reasonable at the posterior, but the Runge effect is amplified at the anterior. However, it is clear from Figure 1(b) that the lower surface is better described than the upper surface. In fact all except the anterior supplementary coordinate are described well by the lower curve. This observation and the symmetry of the nematode prompted the use of that part of the curve from the anterior supplementary coordinate (1, 0) to the first of Cobb's coordinates (0.001, −0.004) to model both the upper and lower boundaries of the nematode (Figure 1(c)). The dotted line underneath Figure 1(c) indicates the portion of the Bézier curve that was used to generate the upper side of the nematode by reflection around the horizontal axis. The remainder of the anterior boundary was completed by linear interpolation from the anterior supplementary coordinate (0,0) to (0.001, 0.004) and to (0.001, −0.004).

## 5. Comparison of Estimates of *p*, *a*, *a*_*s*_, and *v*

**Figure 2:**
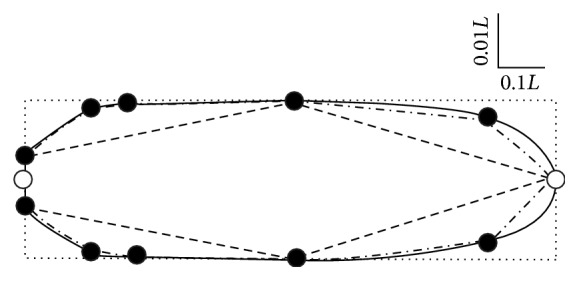
Comparison of the boundaries on which the estimates in Table 1 are based. The Bézier (—) and geometric (-·-·-·-) representations are those we describe. The Tsalolikhin (- - - -) and cylindrical (……) approximations have been reported previously. The morphometric data for* A. antarcticus* reported by Cobb [21] (●) and two supplementary coordinates (○, (0, 0) and (1, 0)) are shown. The vertical and horizontal dimensions differ in scale by a factor of 10 relative to the overall length of the nematode (*L* = 0.6 mm).

The geometric and Bézier representations of* A. antarcticus* described here are bounded by the cylindrical model and they enclose Tsalolikhin's model (2) completely (Figure 2). As (2) can be rewritten as

(31) $$\begin{array}{c} V_{T}=\frac{1}{2}\frac{\pi L}{3}\left[\;\left(\;\frac{d^{2}+dD+D^{2}}{4}\;\right)+\frac{D^{2}}{4}\;\right], \end{array}$$

it is apparent that (2) represents an average of the volumes of a cone (diameter *D* and length *L*) and a conical frustum (length *L*, diameters *D* and *d*). Implicit in this geometrical approach is the assumption that *D* is located at the midpoint of a nematode which gets thinner towards blunt and pointed ends (presumably the anterior and posterior, resp.). Of course, this is equivalent to (15) for *n* = 2, which might be the case for reports of the de Man indices (3).

The geometrical model represents the convex hull of **C** (Figure 2), which necessarily provides a minimum estimate of *p* and *a*. The overestimation inherent in the cylindrical estimate and the underestimation arising from Tsalolikhin's model (2) are clear (Figure 2). To quantify this, the coordinates for* A. antarcticus* (28) were used to calculate *p*, *a*, *a*_*s*_, and *v* (or the corresponding values from the Bézier representation (24)) (Table 1). As would be expected from Figure 2, the Bézier and geometrical representations yield estimates that are similar and lie between those of the cylindrical and Tsalolikhin's (2) models. Arbitrarily taking the geometrical representation as a reference, the cylindrical approach yields a volume 36% larger and (2) yields a value that is 44% smaller (Table 1). Even the value obtained from Andrássy's equation is 9% larger than the geometrical estimate, whereas the Bézier estimate is only 3.5% larger (Table 1).

In the case of* A. antarcticus* the error in *V*_*T*_ arising just from the assumption that the point of greatest diameter is at *L*/2 may be small. Nevertheless, it is instructive to consider a variant of (2) in which the weighting (*λ*) between the conical posterior and an anterior conical frustum can be varied. To do this we write (2) as

(32) VTλπL3λd2+dD+D24+1−λD24=πL3λd2+dD4+D24,

and the error arising from the midlength assumption is

(33) $$\begin{array}{c} \varepsilon_{T}=\frac{V_{T}-V_{T}\left(\lambda \right)}{V_{T}\left(\lambda \right)}=\frac{1}{2}\frac{1-2\lambda }{\lambda +D^{2}/\left(d^{2}+dD\right)}. \end{array}$$

Applying (33) to the values for* A. antarcticus*, in which the widest point is located at 0.51*L* rather than 0.5*L*, yields *ε*_*T*_ = −0.0036, so volume is only slightly underestimated, but Cobb [21] reports data for other species for which *λ* is larger. For example, for* Monhystera polaris d* = 1.5, *D* = 3.4, and *λ* = 0.64, so *ε*_*T*_ = −0.063 which would be a significant underestimate for some purposes, even without any consideration of the other coordinates Cobb [21] reports.

## 6. Conclusion

Estimates of nematode volume are usually made using the cylindrical, Andrássy, and Tsalolikhin approximations. The latter provides an underestimate and the others provide overestimates of nematode size, but they rely on a small number of coordinates. However, the more detailed data available from the Cobb ratios can be used to make better estimates based on the simple geometric or a Bézier approach that we describe. The cylindrical and Tsalolikhin approximations are particular cases of the more general geometric estimate that represents the minimum size of the nematode. The geometric estimate can be extended to incorporate more coordinate data. These approaches could easily be incorporated into appropriate image analysis software [33–36].

## Figures and Tables

**Table 1:** Comparison of estimates of *p*, *a*, *a*_*s*_, and *v* for *A. antarcticus* [21] obtained using the cylindrical, Andrássy (1), and Tsalolikhin (2) approximations [15, 16] and those reported here (12)–(15) and (24). The estimates from the Bézier representation were confirmed numerically using (24).

	*p* or *P* (m)	*a* or *A* (m^2^)	*a*_*s*_ or *S* (m^2^)	*v* or *V* (m^3^)
cylinder^a^	1.229 × 10^−3^	8.64 × 10^−9^	2.75 × 10^−8^	9.77 × 10^−14^
Andrássy^b^	—	—	—	7.82 × 10^−14^
Tsalolikhin^a^	1.188 × 10^−3^	5.04 × 10^−9^	1.58 × 10^−8^	3.98 × 10^−14^
This work				
Geometrical	1.204 × 10^−3^	7.13 × 10^−9^	2.24 × 10^−8^	7.15 × 10^−14^
Bézier	1.208 × 10^−3^	7.54 × 10^−9^	2.26 × 10^−8^	7.40 × 10^−14^

^a^The expressions for *p*, *a*, and *a*_*s*_ for these two approaches are obvious and are not reproduced here.

^b^It is unclear how Andrássy's approach can be applied to the estimation of *p*, *a*, and *a*_*s*_.

## References

[1] Muhamad N., Walker L. R., Pedley K. C., Simcock D. C., Brown S. (2012). The initial kinetics of NH_3_/NH_4_
^+^ efflux from L_3_
*Teladorsagia circumcincta*. *Parasitology International*.

[2] Muhamad N., Simcock D. C., Pedley K. C., Simpson H. V., Brown S. (2011). The kinetic properties of the glutamate dehydrogenase of *Teladorsagia circumcincta* and their significance for the lifestyle of the parasite. *Comparative Biochemistry and Physiology B: Biochemistry and Molecular Biology*.

[3] Sims S. M., Magas L. T., Barsuhn C. L., Ho N. F. H., Geary T. G., Thompson D. P. (1992). Mechanisms of microenvironmental pH regulation in the cuticle of *Ascaris suum*. *Molecular and Biochemical Parasitology*.

[4] Simcock D. C., Brown S., Neale J. D., Przemeck S. M. C., Simpson H. V. (2006). L_3_ and adult *Ostertagia* (*Teladorsagia*) *circumcincta* exhibit cyanide sensitive oxygen uptake. *Experimental Parasitology*.

[5] Stoscheck C. M. (1990). Quantitation of protein. *Methods in Enzymology*.

[6] Sims S. M., Ho N. F. H., Geary T. G. (1996). Influence of organic acid excretion on cuticle pH and drug absorption by *Haemonchus contortus*. *International Journal for Parasitology*.

[7] Hopper B. E., Cairns E. J. (1959). *Taxonomic Keys to Plant, Soil and Aquatic Nematodes*.

[8] McMurtry L. W., Donaghy M. J., Vlassoff A., Douch P. G. C. (2000). Distinguishing morphological features of the third larval stage of ovine *Trichostrongylus* spp.. *Veterinary Parasitology*.

[9] van Wyk J. A., Cabaret J., Michael L. M. (2004). Morphological identification of nematode larvae of small ruminants and cattle simplified. *Veterinary Parasitology*.

[10] Hong C., Timms B. J. (1989). Host-dependent variation in the morphology of female *Ostertagia circumcincta* (Stadelmann, 1894) Ransom, 1907, a nematode parasite of sheep. *Systematic Parasitology*.

[11] Dikmans G., Andrews J. S. (1933). A comparative morphological study of the infective larvae of the common nematodes parasitic in the alimentary tract of sheep. *Transactions of the American Microscopical Society*.

[12] Nguyen K. B., Smart G. C. (1995). Morphometrics of infective juveniles of *Steinernema* spp. and *Heterorhabditis bacteriophora* (Nemata: Rhabditida). *Journal of Nematology*.

[13] Qiu L., Bedding R. (1999). A rapid method for the estimation of mean dry weight and lipid content of the infective juveniles of entomopathogenic nematodes using image analysis. *Nematology*.

[14] Rau G. J., Fassuliotis G. (1970). Equal-frequency tolerance ellipses for population studies of *Belonolaimus longicaudatus*. *Journal of Nematology*.

[15] Andrássy I. (1956). Die rauminhalts- und gewichtsbestimmung der fadenwürmer (Nematoden). *Acta Zoologica Academiae Scientiarum Hungaricae*.

[16] Holovachov O. (2006). *Morphology and Systematics of the Order Plectida Malakhov, 1982 (Nematoda)*.

[17] de Man J. G. (1876). Onderzoekingen over vrij in de aarde levende nematoden. *Tijdschrift der Nederlandsche Dierkundige Vereeninging*.

[18] de Man J. G. (1881). Die einheimischen, frei in der reinen Erde und im süssen Wasser lebenden Nematoden. *Tijdschrift der Nederlandsche Dierkundige Vereeninging*.

[19] Cobb N. A. (1890). *A Nematode Formula*.

[20] Van Cleave H. J. (1948). Expanding horizons in the recognition of a phylum. *The Journal of Parasitology*.

[21] Cobb N. A. (1914). *Antarctic Marine Free-living Nematodes of the Shakleton Expedition. Contributions to a Science of Nematology I*.

[22] Goodchild C. G., Irwin G. H. (1971). Occurrence of nematodes *Rhabditis anomala* and *R. pellio* in oligochaetes *Lumbricus rubellus* and *L. terrestris*. *Transactions of the American Microscopical Society*.

[23] Eveland L. K., Fujino T., Fried B. (1990). Scanning electron microscopical observations of *Pellioditis pellio* (Nematoda: Secernentia). *Transactions of the American Microscopical Society*.

[24] Jensen P. (1979). Nematodes from the brackish waters of the southern archipelago of Finland. Benthic species. *Annales Zoologici Fennici*.

[25] Escuer M., Palomo A., Bello A. (1990). The genus *Ogma* Southern, 1914 (Nematoda: Criconematidae) in the Iberian Peninsula. *Nematologica Mediterranea*.

[26] Stewart A. C., Nicholas W. L. (1994). New species of Xylidae (Nematoda: Monhysterida) from Australian ocean beaches. *Invertebrate Taxonomy*.

[27] Fonseca G., Decraemer W., Vanreusel A. (2006). Taxonomy and species distribution of the genus *Manganonema* Bussau, 1993 (Nematoda: Monhysterida). *Cahiers de Biologie Marine*.

[28] Fracker S. B. (1914). Variation in Oxyurias: its bearing on the value of a nematode formula. *Journal of Parasitology*.

[29] Robinson A. F. (1984). Comparison of five methods for measuring nematode volume. *Journal of Nematology*.

[30] Engels H. (1986). A least squares method for estimation of Bezier curves and surfaces and its applicability to multivariate analysis. *Mathematical Biosciences*.

[31] Apostol T. M. (1967). *Calculus*.

[32] Runge C. (1901). Über empirische funkionen und die interpolation zwischen äquidistanten ordinaten. *Zeitschrift für Mathematik und Physik*.

[33] Atkinson H. J., Urwin P. E., Clarke M. C., McPherson M. J. (1996). Image analysis of the growth of *Globodera pallida* and *Meloidogyne incognita* on transgenic tomato roots expressing cystatins. *Journal of Nematology*.

[34] Das S., Wesemael W. M. L., Perry R. N. (2011). Effect of temperature and time on the survival and energy reserves of juveniles of *Meloidogyne* spp.. *Agricultural Science Research Journal*.

[35] Tsibidis G. D., Tavernarakis N. (2007). *Nemo*: a computational tool for analyzing nematode locomotion. *BMC Neuroscience*.

[36] Otify Y. Z. (2012). Movable computer ruler (MCR): a new method for measuring the size of *Toxoplasma gondii* cysts, tachyzoites and other selected parasites. *Experimental Parasitology*.

